# Analysis of the Bile Salt Export Pump (*ABCB11*) Interactome Employing Complementary Approaches

**DOI:** 10.1371/journal.pone.0159778

**Published:** 2016-07-29

**Authors:** Susanne Przybylla, Jan Stindt, Diana Kleinschrodt, Jan Schulte am Esch, Dieter Häussinger, Verena Keitel, Sander H. Smits, Lutz Schmitt

**Affiliations:** 1 Institute of Biochemistry, Heinrich-Heine-University Düsseldorf, Düsseldorf, Germany; 2 Department of Gastroenterology, Hepatology and Infectious Diseases, University Hospital, Heinrich-Heine-University Düsseldorf, Düsseldorf, Germany; 3 Department of General, Visceral and Pediatric Surgery, University Hospital, Heinrich Heine University Düsseldorf, Düsseldorf, Germany; Hungarian Academy of Sciences, HUNGARY

## Abstract

The bile salt export pump (BSEP, *ABCB11*) plays an essential role in the formation of bile. In hepatocytes, BSEP is localized within the apical (canalicular) membrane and a deficiency of canalicular BSEP function is associated with severe forms of cholestasis. Regulation of correct trafficking to the canalicular membrane and of activity is essential to ensure BSEP functionality and thus normal bile flow. However, little is known about the identity of interaction partners regulating function and localization of BSEP. In our study, interaction partners of BSEP were identified in a complementary approach: Firstly, BSEP interaction partners were co-immunoprecipitated from human liver samples and identified by mass spectrometry (MS). Secondly, a membrane yeast two-hybrid (MYTH) assay was used to determine protein interaction partners using a human liver cDNA library. A selection of interaction partners identified both by MYTH and MS were verified by *in vitro* interaction studies using purified proteins. By these complementary approaches, a set of ten novel BSEP interaction partners was identified. With the exception of radixin, all other interaction partners were integral or membrane-associated proteins including proteins of the early secretory pathway and the bile acyl-CoA synthetase, the second to last, ER-associated enzyme of bile salt synthesis.

## Introduction

One of the liver’s major functions is the production of bile. During digestion bile facilitates the absorption of lipids and fat-soluble vitamins. Furthermore, it serves as the main route for excretion of cholesterol and lipophilic waste products.

The bile salt export pump (BSEP, *ABCB11*) is an ATP binding cassette (ABC)-transporter located in the apical membrane of hepatocytes, which is indispensable for bile flow, as it translocates conjugated bile acids from the cell lumen into the bile canaliculus, driving bile salt-dependent bile flow [1]. At the canalicular membrane BSEP is accompanied by other ABC-transporters, which transport additional bile components, such as the multidrug resistance protein 3 (MDR3, *ABCB4*), which is a lipid floppase specific for the phosphatidylcholine family, and the cholesterol transporter ABCG5/8 [2]. These three compounds, the main organic constituents of bile, form mixed micelles in the canaliculus, which mitigate the toxic detergent effect of bile salts on the surrounding hepato- and cholangiocellular membranes and thereby preserve their integrity [3,4].

Dysfunction of BSEP with respect to expression, trafficking or function is often a molecular determinant of cholestasis. Reduced expression of BSEP at the canalicular membrane or impaired activity leads to accumulation of bile salts in the hepatocyte, which is a cause for benign recurrent or progressive familial intrahepatic cholestasis type 2 (BRIC2 and PFIC2, respectively) [5,6]. Due to their detergent character, increased levels of bile salts lead to cellular damage, while their function as signal molecules impinges on cell metabolism at lower concentrations.

Due to its significance in cholestasis development, many aspects of expression as well as the functional regulation of BSEP have been extensively studied [2,7]. In this field, one less studied aspect is the posttranslational regulation of BSEP by protein-protein interaction (PPI), which is, among other functions, a prerequisite for efficient trafficking and localization of BSEP to the canalicular membrane. Some PPIs, which influence the abundance of the ABC-transporter in the apical membrane have already been identified. For example, the adaptor protein complex 2 (AP-2) and HAX-1 take part in the endocytic retrieval from the canalicular membrane and the myosin II regulatory light chain (MLC2) has been shown to influence anterograde trafficking of the transporter to the apical membrane [8–10]. PPI also plays a role in stabilization of membrane proteins by tethering them to the cytoskeleton. One example is the multidrug-resistance-associated protein 2 (MRP2, *ABCC2*), which depends on scaffolding and membrane-cytoskeletal cross-linking proteins such as the sodium-hydrogen exchanger regulatory factor-1 (NHERF1/EBP50), radixin and ezrin for efficient localization to the apical membrane [11–14]. Additionally, PPI can directly influence the activity of an interaction partner as shown recently for MDR3 (*ABCB4*). Here, the interaction with a scaffold protein regulates trafficking and cell surface expression [15]. This emphasizes that more detailed information on the interaction network of BSEP is required to understand how its trafficking and activity are regulated.

In this study, full-length, human BSEP was used in a membrane yeast two-hybrid (MYTH) screen [16] using a liver cDNA library to identify previously unknown interaction partners. In parallel, proteins associated with BSEP were identified by tandem mass spectrometry (MS/MS) after co-immunoprecipitation (co-IP) from human liver samples. As a third line of interaction studies, putative interaction partners were verified by *in vitro* pull-down assays.

## Materials and Methods

### Yeast strain

The *Saccharomyces cerevisiae* strain NMY51 was used for the membrane yeast two-hybrid assay (Dualsystems Biotech, Schlieren, Switzerland; MATa his3Δ200 trp1-901 leu2-3,112 ade2 LYS2::(lexAop)_4_ -HIS3 ura3::(lexAop)_8_–lacZ ade2::(lexAop)_8_ -ADE2 GAL4). Yeast cells were transformed using the lithium acetate method as described in Gietz *et al*. [17].

### Construction of plasmids for the MYTH screen

For the bait construct full-length human BSEP cDNA [6] was cloned into the pBT3-C vector by homologous recombination. pBT3-C was linearized with the restriction endonucleases NcoI and SfiI. BSEP cDNA was amplified by PCR with the following primer pair 5’-CAAATACACACACTAATCTAGACGGCCATTAATGTCTGACTCAGTAATTCTTCGAAGTATAAAG-3’ and 5’-CTTGATATCGAATTCCTGCAGATATACCCATGACTGATGGGGGATCCAGTGGT-3’. Homologous recombination was performed in the yeast strain NMY51 plated on synthetic defined drop-out medium (SD) lacking leucine. Positive clones were selected by colony PCR. After plasmid isolation and transformation of *E*. *coli* DH5α the construct was verified by sequencing.

### MYTH screen

The MYTH assay was carried out as described in the DUALhunter manual (Dualsystems Biotech). Briefly, the yeast strain NMY51 was transformed with the bait construct pBT3-C-BSEP and the functionality of the system with BSEP as bait was assessed employing the recommended controls. Following that, the bait was tested for self-activation with the empty prey vector pPR3-N. To screen for interaction partners NMY51 was transformed with the bait construct and subsequently with 36 μg of a human adult liver NubG-X cDNA library (Dualsystems Biotech; 1.5x10^6^ independent clones) for complete coverage. Clones grown on SD medium lacking leucine, tryptophan and histidine (SD-LWH) were re-plated on SD medium lacking in addition adenine and supplemented with 40 μg/ml X-Gal (SD-LWHAdX). Plasmids of blue colonies were isolated and amplified in *E*. *coli* DH5α. Yeast cells harboring the pBT3-C-BSEP plasmid were retransformed with the prey plasmids to confirm the interaction. Interaction partners were checked for false positives by a bait dependency test with the SV40 large T antigen fused to an Ost4p membrane anchor as unrelated bait (DUALhunter manual, control plasmid pDHB1-largeT). Remaining candidates were sequenced and identified with the basic local alignment search tool (BLAST) [18]. MYTH controls were performed at least in duplicate.

### Cloning of putative interaction partners for production in *E*. *coli*

cDNA of potential interaction partners identified in the MYTH screen were cloned into the pET-51b(+) vector (EMD Biosciences, Inc., Darmstadt, Germany) for expression in *E*. *coli*. cDNA was amplified by PCR with the addition of ZraI and KpnI restriction endonuclease sites and inserted into pET-51b(+) via these restriction sites. All constructs were verified by DNA sequencing.

### Production of putative interaction partners in *E*. *coli*

Full-length radixin and radixin_1-318_ were produced in *E*. *coli* BL21 (DE3), the bile acyl-CoA synthetase (BACS) in *E*. *coli* Rosetta (DE3) pLysS. LB medium (10 g/l Tryptone/Peptone from Casein, 5 g/l yeast extract, 5 g/l NaCl) or in the case of radixin and radixin_1-318_ LBN (10 g/l Trypton/Pepton from Casein, 2 g/l glucose, 29.2 g/l NaCl) was inoculated to an OD_600_ of 0.09 and grown to an OD_600_ of 0.6 at 37°C and 180 rpm. Protein production was induced by addition of 0.5 mM IPTG. The proteins were produced at 18°C for 20 h. After cell harvest (3000 *g*, 20 min, 4°C) the sediment was suspended in lysis buffer (50 mM sodium phosphate pH 7, 100 mM NaCl, 1 mM EDTA, 20% (w/v) glycerol) supplemented with protease inhibitor cocktail (Roche, Basel, Switzerland) and lysed by two passages through the Microfluidizer M-110P (Microfluidics, Westwood, MA) at 1.3 kbar. The lysate was cleared by centrifugation at 4000 *g*, 15 min, 4°C followed by centrifugation at 15000 *g*, 30 min, 4°C.

### Purification of interaction partners

Protein interaction partners were purified by affinity chromatography. *E*. *coli* cell lysate was applied to Strep-Tactin resin (iba GmbH, Göttingen, Germany) by gravity flow and washed with buffer (50 mM HEPES pH 7, 150 mM NaCl, 1 mM EDTA). Protein was eluted in elution buffer (50 mM HEPES pH 7, 150 mM NaCl, 1 mM EDTA, 2.5 mM desthiobiotin) and concentrated with Amicon centrifugal filter units with a molecular weight cut-off of 10 kDa (Merck KGaA, Darmstadt, Germany). Purified protein was flash frozen and stored at -80°C.

### Expression of BSEP

BSEP was produced in the methylotrophic yeast *Pichia pastoris* (*Komagataella pastoris*) as previously published in Ellinger *et al*. [19]. In brief, *Pichia pastoris* X-33 (Life Technologies, Carlsbad, CA) was transformed with the construct pSGP18-2μ-BSEP. The yeast was fermented according to the Invitrogen Pichia Expression Kit manual in a 15 liter table-top glass fermenter (Applikon Biotechnology, Schiedam, the Netherlands) in 5 l of basal salt medium with addition of 500 ml of 50% (v/v) glycerol. Feeding 500 ml of methanol during 28 h induced protein production. Approximately 800 g of wet cell mass was flash-frozen in liquid nitrogen and stored at -80°C until further use.

### Purification of BSEP

*Pichia pastoris* cells were suspended in homogenization buffer (50 mM Tris pH 8, 50 mM NaCl, 0.33 M Sucrose, 1 mM EDTA pH 8, 1 mM EGTA pH 8, 0.1 M 6-aminohexanoic acid, 1 mM DTT) supplemented with protease inhibitor cocktail tablets (Roche). Cell disruption was performed with the Microfluidizer M-110P (Microfluidics) in three passes at 2 kbar. Cell debris was sedimented by differential centrifugation (1500 *g*, 15 min, 4°C; 14000 *g*, 20 min, 4°C). Crude membranes were obtained by ultracentrifugation at 120000 *g* for 1 hour at 4°C and suspended in membrane buffer (50 mM Tris pH 8, 50 mM NaCl, 20% glycerol). After a second ultracentrifugation step, the membranes were resuspended in membrane buffer to a protein concentration of 10–20 mg/ml. Membranes equivalent to 30 g of wet cell weight were diluted to a protein concentration of 5 mg/ml as determined by Pierce Coomassie Plus Assay (Thermo Fisher Scientific Inc., Rockford, IL). Fos-choline 16 (Anatrace, Maumee, OH) was added to a concentration of 1% (w/v) and proteins solubilized with rotation at 4°C for 45 min. Aggregates were sedimented by ultracentrifugation at 100000 *g*, 40 min, 4°C. The supernatant was applied to 1 ml of Calmodulin Affinity Resin (Agilent Technologies, Santa Clara, CA) and incubated with light agitation at 4°C for 30 min. The resin was washed with CBP binding buffer (50 mM Tris pH 8, 150 mM NaCl, 5 mM β-mercaptoethanol, 1 mM magnesium acetate, 2 mM calcium chloride, 15% (w/v) glycerol, 0.022% (w/v) DDM). BSEP was eluted with CBP elution buffer (50 mM Tris pH 8, 150 mM NaCl, 5 mM β-mercaptoethanol, 2 mM EGTA, 15% (w/v) glycerol, 0.022% (w/v) DDM), stored at 4°C and used within the next 48 h. For removal of the affinity tags 200 μg of BSEP were incubated with 2 units of HRV 3C Protease (Thermo Scientific, Rockford, IL) for 4 h at 4°C. Protease and affinity tags were removed by incubation with 50 μl of Ni-NTA Agarose (Qiagen, Hilden, Germany) for 30 min at 4°C.

### Pull-down assay of BSEP with interactions partners

For pull-down assays, interaction partners were immobilized on a Strep-Tactin resin. After 30 min incubation at 4°C and washing with pull-down buffer (50 mM sodium phosphate pH 7, 150 mM NaCl, 20% (w/v) glycerol, 0.022% (w/v) DDM) detergent-solubilized, purified BSEP was added for another 30 min at 4°C. Complexes were eluted after 5 times washing and analyzed by immunoblotting. For detection of BSEP, the monoclonal F-6 antibody (Santa Cruz Biotechnology, Dallas, TX, AB_2242103) was used, the Strep-tag II was detected by anti Strep-tag II mouse monoclonal antibody (Merck KGaA, Darmstadt, Germany, AB_10807650). Horseradish peroxidase-conjugated goat monoclonal secondary antibody was purchased from Dianova (Hamburg, Germany). Pull-down assays were replicated at least three times.

### Immunoprecipitation of BSEP from human liver

This study was performed according to the guidelines of the declaration of Helsinki. Written consent was obtained from all patients, and all associated studies on excess material were approved by the local ethics committee (study no. 2852 approved by the local ethics committee of the medical faculty of Heinrich Heine University Düsseldorf). Human liver samples were obtained from the noncancerous resection margin during liver metastasis resection and were immediately processed. All steps were carried out on ice or at 4°C unless stated otherwise. Liver tissue was cut into small pieces and lysed in homogenization buffer (50 mM Tris/HCl pH 7.4, 250 mM sucrose) with eight strokes in a tight-fitting dounce homogenizer. The crude homogenate was centrifuged for 10 min at 1000 *g*, and afterwards for 10 min at 3000 *g*. 20 μl of the wet pellet were solubilized in 1 ml IP solubilization buffer (1% Triton X-100 or digitonin in 50 mM HEPES pH 7.5, 100 mM NaCl, 1 mM EDTA, 1 mM EGTA, 10% glycerol, Roche protease inhibitor cocktail (EDTA-free)) overnight with slow overhead inversion. Insoluble material was removed by centrifugation at 13000 *g* for 10 min. For immunoprecipitation the monoclonal BSEP antibody (F-6) was used and naïve mouse IgG_2a_ served as control (Santa Cruz Biotechnology, Dallas, TX). 2 μg of antibody were added to 20 μl of protein A/G+ agarose slurry (Santa Cruz Biotechnology) and the mixture was incubated for 10 min at room temperature with rotation. The preloaded agarose was washed three times with IP solubilization buffer. 1 ml of the solubilization supernatant was added to agarose-bound antibody, samples were incubated for 4 h with slow overhead inversion and centrifuged for 1 min at 1000 *g*. The resulting pellet was washed three times with IP solubilization buffer. Remaining buffer was removed by aspiration and pellets were stored at -20°C. For further analysis, the agarose pellet was incubated in 64 μl 3% (w/v) SDS for 20 min, followed by addition of 16 μl 5x reducing sample buffer (100 mM Tris/HCl pH 6.8, 3.2% (w/v) SDS, 40% (v/v) glycerol, 0.02% bromophenol blue, 10% β-mercaptoethanol). After incubation at 65°C for 15 min samples were spun down briefly and 40 μl were subjected to SDS-PAGE and immunoblotting. Detection was performed with the following antibodies: polyclonal anti-BSEP K24, which was raised in rabbit against the C-terminal 13 amino acids as described previously [20], mouse monoclonal anti-Radixin (GeneTex, Irvine, CA, AB_10624814), mouse monoclonal anti-Adaptin α (BD Biosciences, Franklin Lakes, NJ, AB_397867) or rabbit polyclonal anti-SLC27A5 (Abcam plc, Cambridge, UK, AB_2190760). These experiments were performed at least twice.

### Mass spectrometry

Following detergent removal, a tryptic digest of the co-IP pellet was subjected to shotgun tandem mass spectrometry. The analysis was carried out on an UHPLC-coupled Orbitrap Elite mass spectrometer by the Molecular Proteomics Laboratory at the Biologisch-Medizinisches Forschungszentrum (BMFZ) of Heinrich-Heine-University Düsseldorf as detailed earlier [21].

### Immunofluorescence staining of liver cryosections and differentiated HepaRG cells

Cryosections were obtained from human liver tissue used as source material for co-IP experiments. HepaRG cells were cultured and differentiated as described [22]. Briefly, the cells were grown for two weeks without subculturing and culture medium exchanged every two days, followed by two weeks under medium supplemented with 2% (v/v) dimethyl sulfoxide. Differentiated cells were harvested and seeded onto glass coverslips before cultivation for 48h in DMSO-containing medium. Both cells and liver cryosections were fixed in ice-cold methanol and blocked in Ultra V block (Thermo). BSEP was stained using either mouse F-6 (Santa Cruz, 1:100) or the rabbit serum K24 [20], BACS and MRP2 were stained using rabbit anti-SLC27A5 (Abcam, 1:100) and mouse M2-III6 (1:25; Alexis, AB_2273479), respectively. Goat anti-mouse-IgG2a-AF488 (AB_2535771) and goat anti-rabbit-IgG-AF546 (AB_2534093, both 1:500, Invitrogen) were used as secondary antibodies and cell nuclei stained with Hoechst 34580. All samples were analyzed by confocal laser-scanning microscopy on a Zeiss LSM 510meta confocal laser-scanning microscope.

## Results

### Three complementary methods identify a novel set of BSEP interaction partners

A comprehensive understanding of the regulation of a protein in regard to function and cellular trafficking requires detailed knowledge of its interactome. Techniques such as yeast two-hybrid approaches, tandem affinity purification or immunoprecipitation, the latter two often combined with mass spectrometry, have been developed and optimized in recent years. However, each of these techniques has its own advantages and disadvantages [23]. For example, in yeast two-hybrid systems the presence of protein tags can prevent interactions or produce false positive results, while mass spectrometry-based approaches lack some sensitivity towards transient interactions. To cope with these limitations and to advance the understanding of BSEP on a molecular level we used three complementary methods to identify interacting proteins of this bile salt transporter.

**Fig 1:**
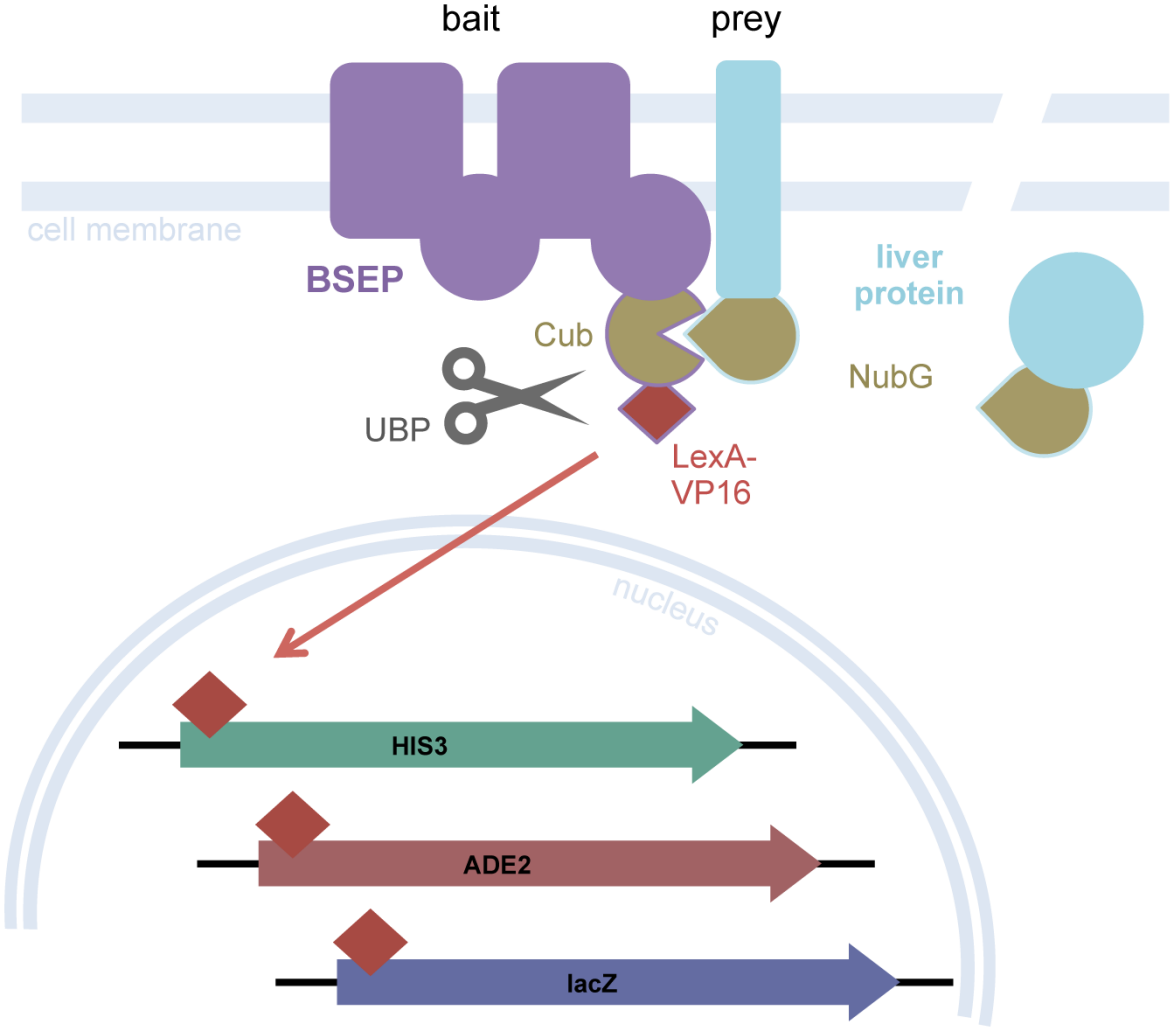
Schematic diagram of the membrane yeast two-hybrid system (MYTH) to screen for interaction partners of the ABC-transporter BSEP. The MYTH system is based on a split-ubiquitin approach. The bait, BSEP, is fused to the C-terminus of ubiquitin (Cub) and a transcription factor (LexA-VP16). The preys are soluble or membrane-associated liver proteins, introduced via a cDNA library. They are fused to the N-terminus of ubiquitin (NubG). Upon interaction of BSEP and the liver protein the ubiquitin moieties, which have a low affinity for each other due to a mutation in the N-terminus, come into close proximity. The reassembled ubiquitin is recognized by endogenous ubiquitin specific proteases (UBP). The transcription factor is cleaved off and activates the reporter genes (*HIS3*, *ADE2*, *lacZ*).

Firstly, the MYTH assay was used to screen for putative interaction partners that interact directly with BSEP. In contrast to conventional yeast two-hybrid systems [23,24] the MYTH system, which has been established by Stagljar *et al*., allows for screening of membrane proteins [16,25]. A schematic diagram of the system is provided in Fig 1. The MYTH assay is based on the re-association of two ubiquitin halves. The protein of interest (bait) is produced in yeast as a fusion protein with the C-terminal half of ubiquitin (Cub) and a transcription factor (LexA-VP16). The putative interaction partners (prey) are fused to the mutated N-terminal moiety of ubiquitin (NubG). Interaction of bait and prey cause the two ubiquitin halves to reconstitute into the so called split-ubiquitin, which is recognized by an endogenous ubiquitin specific protease. The transcription factor is released and activates three reporter genes, which enable yeast to grow on selective media [16].

For our study the assay was established with full-length, human BSEP as bait. BSEP cDNA was cloned into the pBT3-C bait vector, which adds the required ubiquitin moiety and transcription factor to the C-terminus of the protein for optimal accessibility of the fusion proteins.

**Fig 2:**
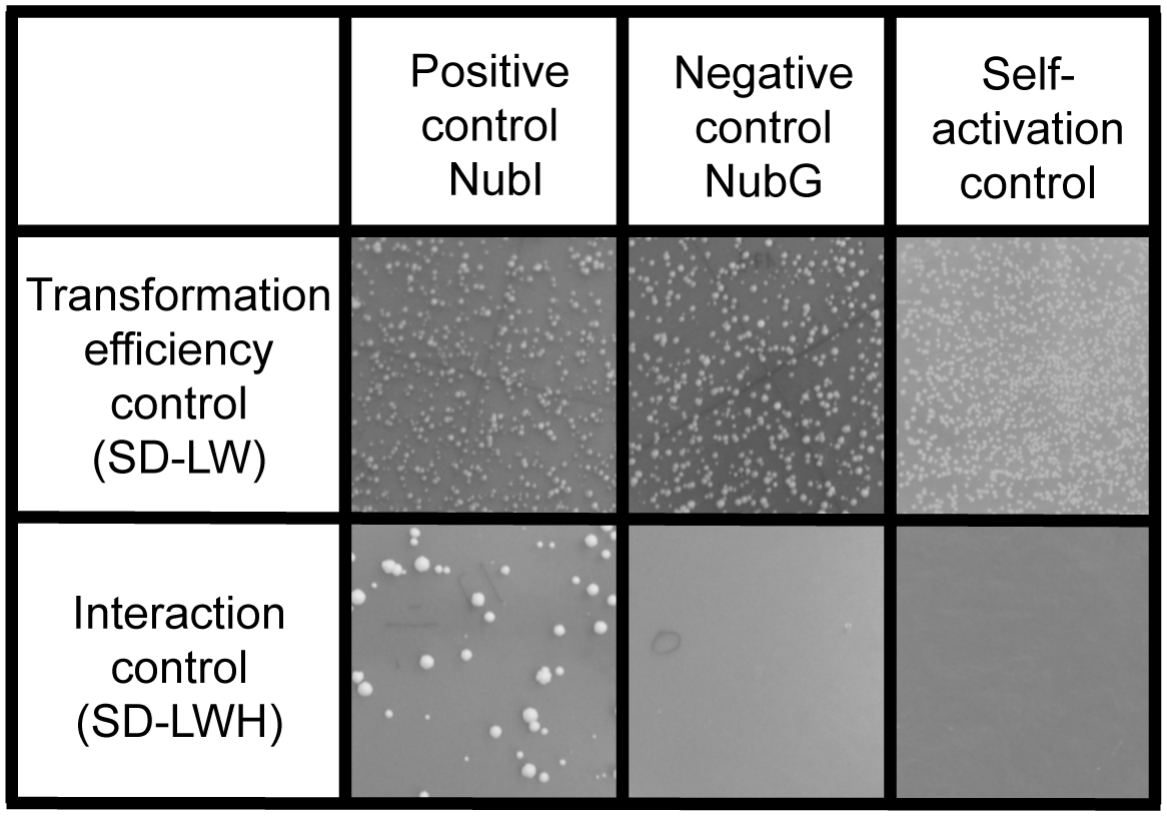
Functional control assay for the membrane yeast two-hybrid with BSEP and self-activation control. The yeast strain NMY51 was transformed with the BSEP bait construct and control plasmids coding either for a nonsense peptide with the NubI (wild-type ubiquitin) or the NubG (mutated ubiquitin) -tag. Due to its affinity to its C-terminal half the NubI moiety activates the system regardless of bait interaction. Both controls show equal transformation efficiency on selective media (SD-LW) for the plasmids (upper panels). The positive control shows yeast growth on selective medium (SD-LWH) due to the affinity of the wild-type ubiquitin moieties. This confirms expression of the BSEP fusion protein. In contrast, the negative control shows no reporter gene activation based on unspecific interaction of BSEP with the NubG-nonsense peptide. Additionally, no self-activation of the MYTH system with BSEP was detected in a library-scale transformation of the empty pPR3-N library vector.

Functionality of the MYTH system with BSEP was ascertained by control assays. The first assay activated the system independently of bait interaction and thereby confirmed BSEP expression (Fig 2). The second control assay tested the BSEP construct for self-activation, which is a common complication in applying the MYTH assay. A low bait expression level, which was indicated for this system in the first control assay, is of advantage. BSEP did not show self-activation in our setup (Fig 2).

To screen for interaction partners the BSEP expressing yeast strain was transformed with a human adult liver cDNA library. This library had a complexity of 1.5x10^6^ independent clones and allowed for the screening of liver proteins or protein fragments of up to 450 amino acids.

As in any genetic screening method, the obtained clones contained a number of false positives. To reduce their amount, reporter gene activation was reconfirmed individually for each prey plasmid and subsequently analyzed in a bait dependency test, where the reporter activation for a prey was compared between BSEP and an unrelated, non-interacting bait. Sequencing and identification of the cDNA fragments by alignment search tools led to elimination of the remaining false positive clones, which occurred due to out of frame readings or gene products from non-relevant compartments. This resulted in the identification of 37 proteins (Table 1).

**Table 1:** Protein and gene names of identified interaction partners of BSEP derived from the MYTH assay.

Protein Name	Gene Symbol	GeneID	GenBank Accession
aldolase A, fructose-bisphosphate	ALDOA	226	NM_184041.2
asialoglycoprotein receptor 2	ASGR2	433	NM_080914.2
B-cell receptor-associated protein 31	BCAP31	10134	NM_001256447.1
BCL2/adenovirus E1B 19kDa interacting protein 3-like	BNIP3L	665	NM_004331.2
bile acyl-CoA synthetase	SLC27A5	10998	NM_012254.2
catechol-O-methyltransferase	COMT	1312	NM_000754.3
CD63 molecule	CD63	967	NM_001780.5
CD99 molecule	CD99	4267	NM_001122898.1
cofilin 1 (non-muscle)	CFL1	1072	NM_005507.2
dolichyl-phosphate mannosyltransferase polypeptide 2, regulatory subunit	DPM2	8818	NM_003863.3
ER membrane protein complex subunit 4	EMC4	51234	NM_016454.2
glutamate receptor, ionotropic, N-methyl D-aspartate-associated protein 1	GRINA	2907	NM_001009184.1
heme oxygenase (decycling) 2	HMOX2	3163	NM_002134.3
immediate early response 3 interacting protein 1	IER3IP1	51124	NM_016097.4
interferon induced transmembrane protein 2	IFITM2	10581	NM_006435.2
interferon induced transmembrane protein 3	IFITM3	10410	NM_021034.2
mitogen-activated protein kinase binding protein 1	MAPKBP1	23005	NM_001265611.1
protein disulfide isomerase family A, member 6	PDIA6	10130	NM_005742.2
radixin	RDX	5962	NM_001260492.1
receptor accessory protein 5	REEP5	7905	NM_005669.4
RER1 retention in endoplasmic reticulum 1 homolog (*S*. *cerevisiae*)	RER1	11079	NM_007033.4
signal peptidase complex subunit 1 homolog (*S*. *cerevisiae*)	SPCS1	28972	NM_014041.3
signal peptidase complex subunit 2 homolog (*S*. *cerevisiae*)	SPCS2	9789	NM_014752.2
signal sequence receptor, gamma (translocon-associated protein gamma)	SSR3	6747	NM_007107.3
sphingomyelin phosphodiesterase 4, neutral membrane (neutral sphingomyelinase-3)	SMPD4	55627	NM_017951.4
translocation associated membrane protein 1	TRAM1	23471	NM_014294.5
transmembrane protein 134	TMEM134	80194	NM_025124.2
transmembrane protein 14A	TMEM14A	28978	NM_014051.3
transmembrane protein 199	TMEM199	147007	NM_152464.1
transmembrane protein 205	TMEM205	374882	NM_001145416.1
transmembrane protein 230	TMEM230	29058	NM_001009925.1
UDP glucuronosyltransferase 1 family, polypeptide A5	UGT1A5	54579	NM_019078.1
unconventional SNARE in the ER 1 homolog (*S*. *cerevisiae*)	USE1	55850	NM_018467.3
VAMP (vesicle-associated membrane protein)-associated protein B and C	VAPB	9217	NR_036633.1
WD repeat domain 83 opposite strand	WDR83OS	51398	NM_016145.3
Yip1 interacting factor homolog A (*S*. *cerevisiae*)	YIF1A	10897	NM_020470.2
ZMYM6 neighbor	ZMYM6NB	100506144	NM_001195156.1

Highlighted proteins have also been identified in the co-immunoprecipitation / MS/MS screen. Five proteins are human homologs of *S*. *cerevisiae* proteins as indicated by the species added in brackets.

Secondly, immunoprecipitation of BSEP from human liver and consecutive analysis of the co-precipitated protein complexes by MS/MS allowed for the identification of proteins associated with the transporter. For solubilization of BSEP we tested two commonly used, mild detergents, Triton X-100 and digitonin. To identify BSEP-specific precipitation, protein frequencies from co-IP with BSEP antibody were compared to the frequencies obtained with a control antibody. Using this strategy, 262 proteins were identified.

**Fig 3:**
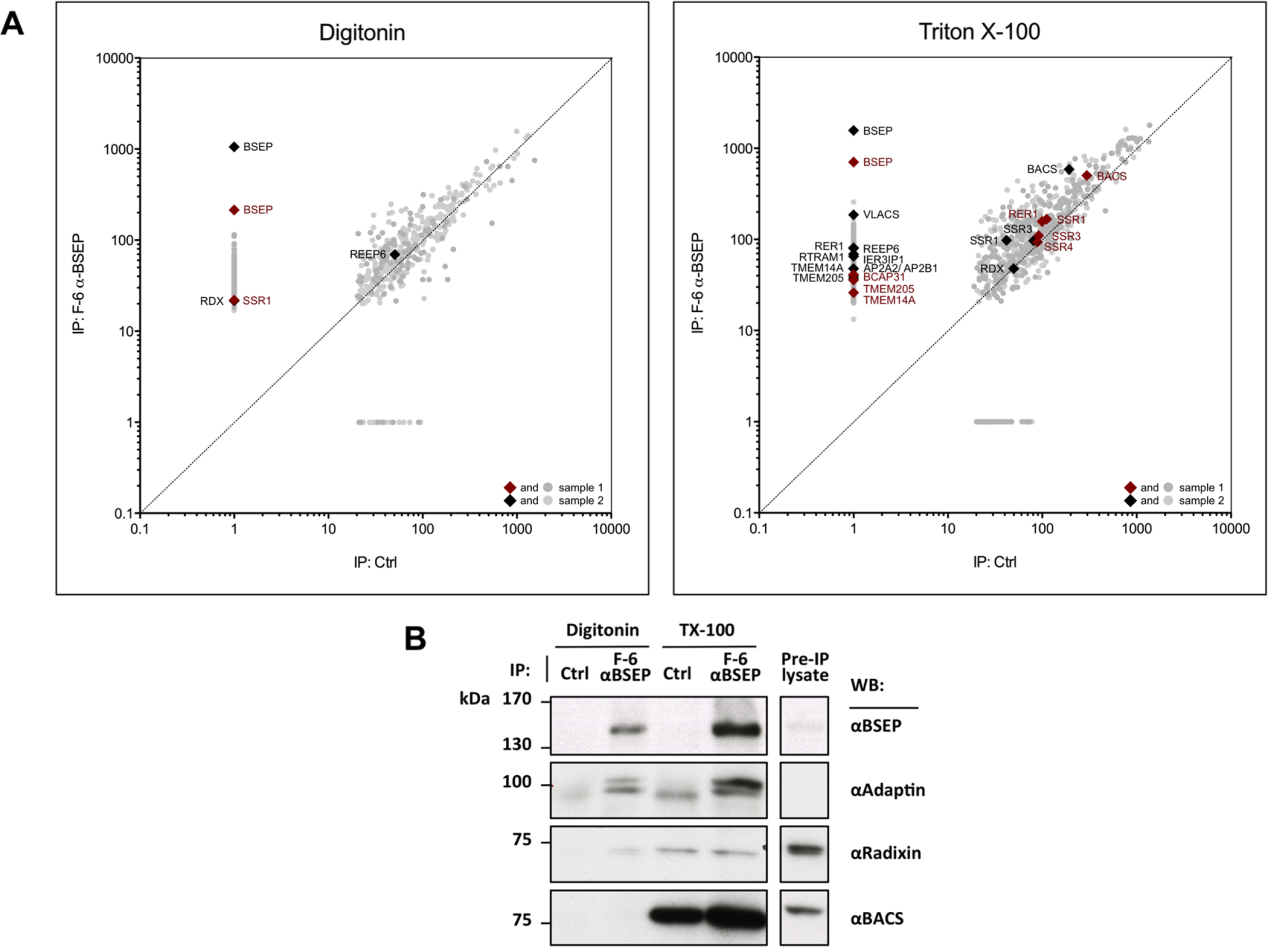
co-IP / MS/MS identifies BSEP interaction partners. **(A)** Immunoprecipitation of BSEP coupled to complex mass spectrometry reveals new interaction partners of BSEP in human liver. Crude canalicular membrane preparations were solubilized in either digitonin or Triton X-100, and immunoprecipitated samples were subjected to MS/MS. For each detergent, protein frequencies from two co-IPs with BSEP antibody are plotted against the respective negative control with naïve mouse IgG. Interaction partners of interest are labeled. For the sake of clarity, proteins found exclusively in either the BSEP or control co-IP are depicted with an MS-score of one instead of zero on the other axis. (**B)** Immunoblot analysis of co-IPs shown inA.

The adaptor protein complex 2 (AP-2) is a known interaction partner of BSEP [9] and was precipitated in this setup, while it was not identified in the MYTH screen. Subunits of the complex were detected by MS/MS after solubilization of canalicular membranes with Triton X-100, while specific AP-2 signals were absent in samples prepared with digitonin (Fig 3A; compare S9 Fig for an assembly of interaction partners derived from MS data only from all four co-IPs). Immunoblots of the samples however showed that subunits of AP-2 were precipitated with BSEP in both detergents (Fig 3B).

**Fig 4:**
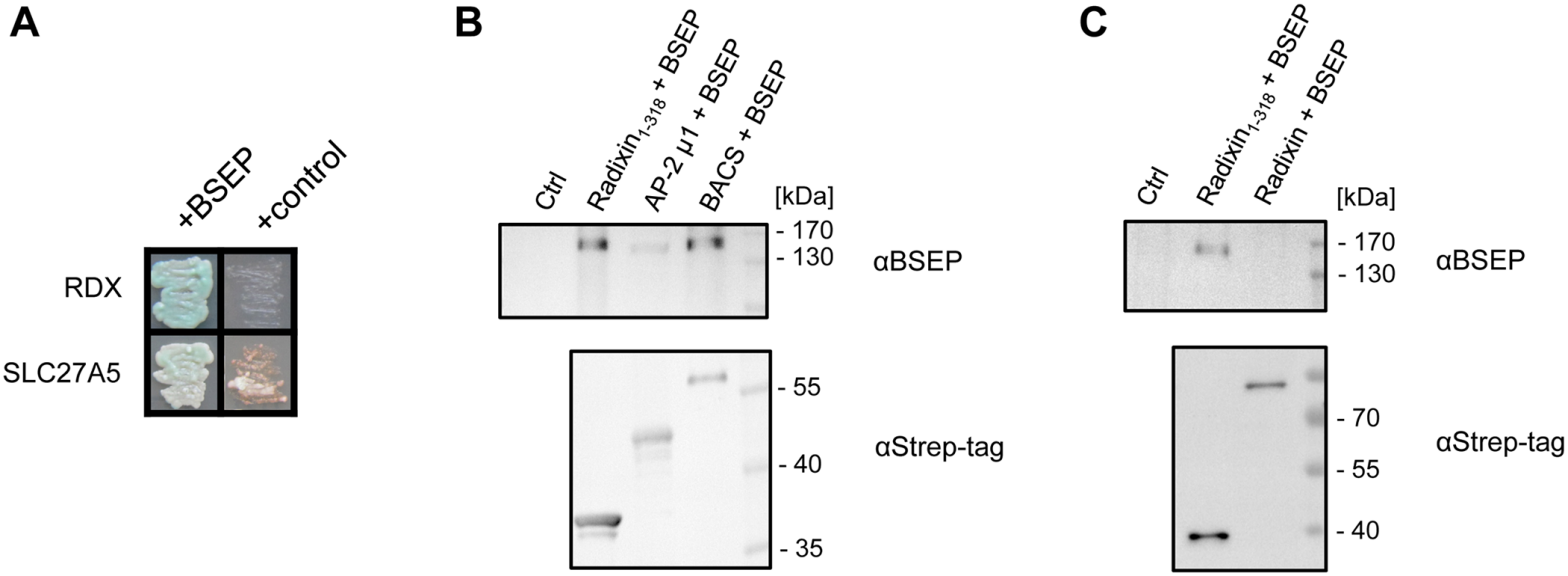
BSEP interacts with radixin and the bile acyl-CoA synthetase. **(A)** MYTH bait dependency test of radixin_1-318_ and BACS_640-690_ against BSEP or a non-interacting control bait. (**B)** Pull-down of BSEP with radixin_1-318_, BACS_77-690_ and AP-2 μ1. Protein interaction partners were immobilized as bait on Strep-Tactin Sepharose and purified BSEP was added. BSEP and the interaction partners were detected by immunoblot analysis with monoclonal antibodies against BSEP or the Strep-tag, respectively. Strep-Tactin Sepharose without bait protein served as negative control. (**C)** Tag-less BSEP can be pulled down with radixin_1-318_, but not with full-length, non-activated radixin. Protein interaction partners were immobilized on Strep-Tactin Sepharose. Purified, tag-less BSEP was added to the beads and the complexes were eluted after washing. BSEP and the interacting proteins were detected by immunoblot analysis with monoclonal antibodies against BSEP or the Strep-tag, respectively.

**Fig 5:**
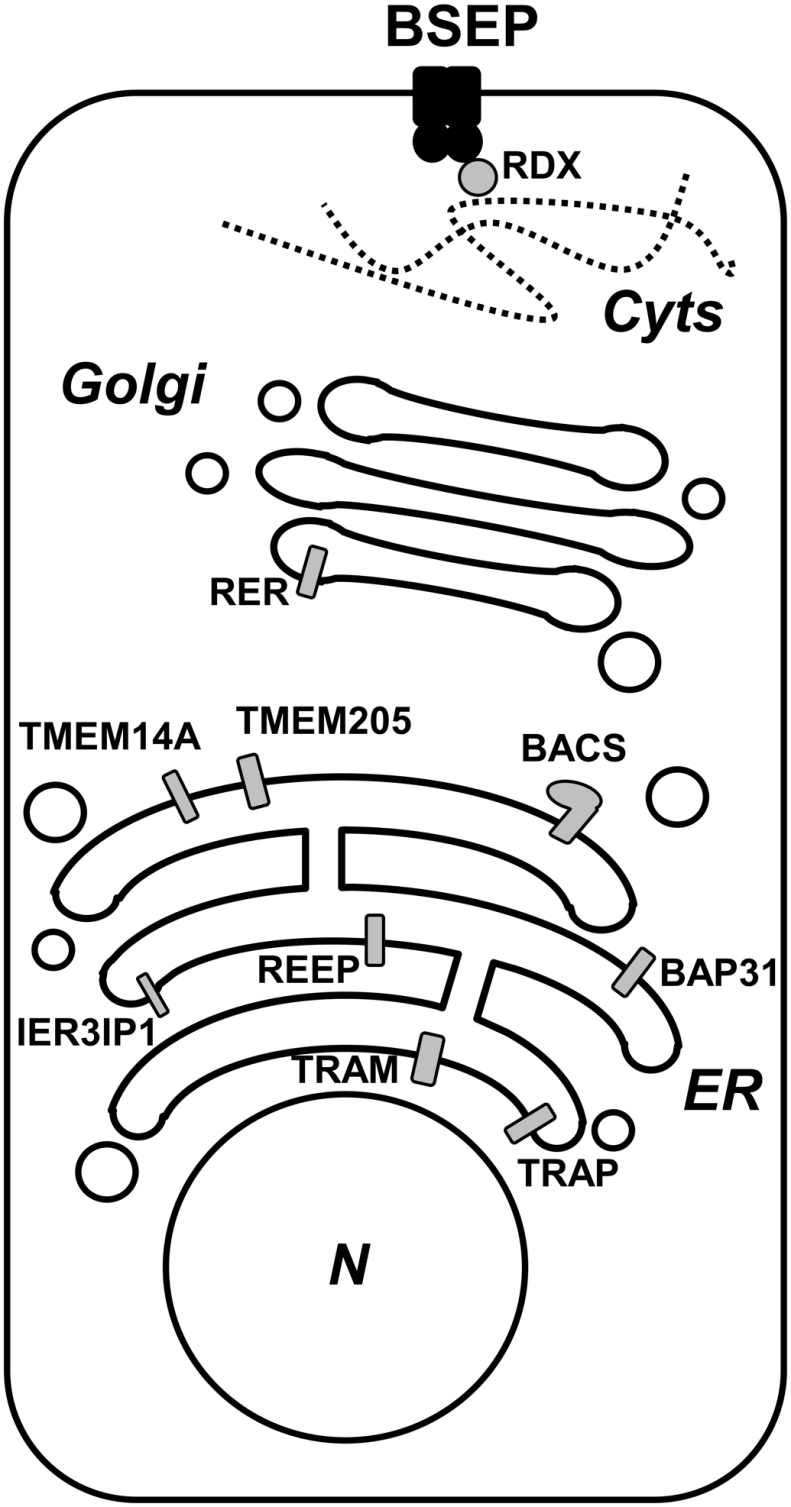
Schematic overview of the subcellular localization of identified BSEP interaction partners. The ten novel interaction partners of BSEP are shown in a cellular context. Depicted are the nucleus (N), endoplasmic reticulum (ER), Golgi apparatus (Golgi) and cytoskeleton at the apical membrane (Cyts).

Thirdly, *in vitro* pull-down analyses were used to confirm a subset of identified interaction partners using recombinant, purified proteins. Here, the alpha subunit of the AP-2 complex as a known interaction partner [9] was able to pull down BSEP (Fig 4B). The subcellular localization of the ten identified BSEP interaction partners that were identified in both screens is depicted in Fig 5. In the following section, we will focus on the proteins that were identified with these complementary methods (Table 2).

**Table 2:** Proteins identified as interaction partners of BSEP in MYTH and co-IP / MS/MS screens.

Protein Name and Gene Symbol	GenBank Accession
B-cell receptor-associated protein 31 (BCAP31)	NM_001256447.1
bile acyl-CoA synthetase (SLC27A5) [SLC27A2]	NM_012254.2
immediate early response 3 interacting protein 1 (IER3IP1)	NM_016097.4
receptor accessory protein 5 (REEP5) [REEP6]	NM_005669.4
retention in endoplasmic reticulum 1 (RER1)	NM_007033.4
radixin (RDX)	NM_001260492.1
signal sequence receptor, gamma (translocon-associated protein gamma) (SSR3) [SSR4]	NM_007107.3
translocation associated membrane protein 1 (TRAM1)	NM_014294.5
transmembrane protein 14A (TMEM14A)	NM_014051.3
transmembrane protein 205 (TMEM205)	NM_001145416.1

Gene names of closely related proteins identified in the co-IP screen are added in square brackets.

### Factors of the early secretory pathway

**Fig 6:**
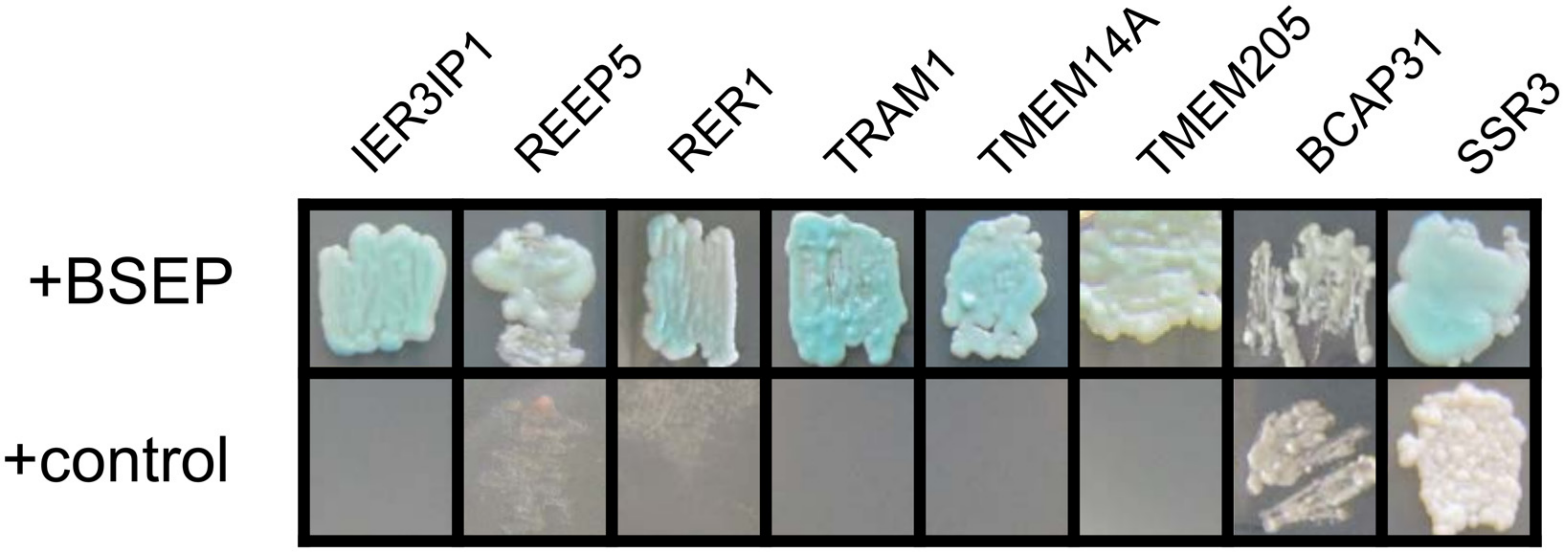
Bait dependency test with BSEP interaction partners in the early secretory pathway. To confirm the interaction with prey proteins from the initial MYTH screen, reporter gene activation was tested individually for each prey against the bait, BSEP, or a non-interacting control bait, large T antigen. The figure shows one representative result for the positively tested preys, which are denoted by their respective gene name.

Nine out of the ten proteins identified in both screens are membrane proteins of the early secretory pathway. All proteins discussed in the following paragraph co-precipitated specifically with BSEP from human liver and required Triton X-100 for solubilization (see Fig 3, S9 Fig and S1 Table). Also, these proteins showed direct interaction with BSEP as seen in the bait dependency test performed after the MYTH screen (Fig 6).

The translocon associated protein (TRAP) is a complex of four subunits involved in membrane protein topogenesis [26]. The two screens identified the gamma subunit (*SSR3*) as a direct interaction partner of BSEP, while the alpha and delta subunit (*SSR1/4*) were found to associate with the transporter in the co-IP / MS/MS screen. While the interaction of TRAP gamma subunit and BSEP displayed a comparatively high background signal in both screens (Fig 6 and S1 Table), the alpha and delta subunits co-precipitated specifically with BSEP (S1 Table, S9 Fig). Taken together, these findings suggest an interaction of the TRAP complex with BSEP.

A second factor connected to the translocation of hydrophobic protein sections is the translocating chain-associated membrane protein 1 (*TRAM1*). TRAM1 has been found to be stimulatory or for some targets even necessary for membrane protein translocation. Furthermore, a role for TRAM in dislocation of proteins from the ER has been postulated [27,28].

Another aspect of trafficking is membrane protein sorting and quality control. Bap31 (*BCAP31*) is an ubiquitous, integral ER membrane protein, which is closely associated with TRAM and involved in protein sorting. Its expression affects a number of membrane proteins, for example CFTR (*ABCC7*) [29]. Some mutations involving *BCAP31* could be connected to liver dysfunction and cholestasis [30,31].

The receptor expression-enhancing proteins (*REEP*) of which proteins 5 and 6 were identified in MYTH and co-IP / MS/MS screen, respectively, are proposed to be ER-shaping proteins which directly interact with cargo proteins to modulate their processing and trafficking [32].

The immediate early response 3 interacting protein 1 (*IER3IP1*) is a less studied, ER-resident protein [33]. Two mutations have been linked to microcephaly and an abnormal amount of apoptosis [34]. Its homology to the yeast protein Yos1 points towards a function in vesicular transport between Golgi and ER.

Retention in endoplasmic reticulum 1 (*RER1*) is an early Golgi membrane protein thought to function as a sorting chaperone, which modulates the fate of several membrane proteins [35]. While RER1 came up in both screens, in the MYTH screen only the C-terminal half of the 196 amino acid protein was encoded on the library plasmid. This part codes for the last transmembrane helix and the cytosolic tail of the protein. Studies of the yeast homolog Rer1 show that cargo recognition occurs via transmembrane domain interaction, while the C-terminus has been shown to bind to the coatomer [36].

There is little information on two further interaction partners identified. Transmembrane protein 14A (*TMEM14A*) was found to localize to or close to ER and mitochondria and ectopic expression resulted in apoptosis suppression [37]. Its molecular function has not yet been elucidated. Transmembrane protein 205 (*TMEM205)* has been identified in endosomes of the liver and other secretory tissue and its expression was linked to increased cisplatin resistance [38]. Current information would support a role of this small membrane protein in secretion or vesicular trafficking.

All identified proteins are candidates that may directly determine the fate of BSEP at different stages along the secretory pathway.

### BSEP interacts with an ER-anchored enzyme

In addition to the factors related to protein sorting and quality control, BSEP showed interaction with an ER-anchored enzyme.

The bile acyl-CoA synthetase (BACS, *SLC27A5*) catalyzes the first step in re-conjugation of bile acids to taurine or glycine [39]. BACS was found to co-precipitate with BSEP as seen in the MS/MS analysis from samples solubilized with Triton X-100 (Fig 3A). With the detergent digitonin corresponding peptides were not found in co-IP samples (S1 Table). Immunoblots of the co-IP samples also showed that BACS is precipitated to a greater degree in the presence of BSEP (Fig 3B). Interestingly, a relative of BACS, the very long-chain acyl-CoA synthetase (VLACS, *SLC27A2*), was specifically co-precipitated with BSEP in Triton X-100 (Fig 3A). BACS was also identified as a BSEP-interacting protein in the MYTH assay (Fig 4A). Here, the C-terminal 50 amino acids were encoded by the library plasmid, a fragment that belongs to the cytosolic part of the enzyme. This fragment shares 50% identity with the related VLACS. Pull-down analyses, as seen in Fig 4B, confirm the interaction of BSEP with BACS lacking its N-terminal membrane anchor (BACS_77-690_). This finding might explain the observation of Wlcek *et al*. [40] who observed a protein dependent influx of taurocholate in the lumen of the rough endoplasmic reticulum.

Since BSEP is localized at the apical, canalicular membrane and BACS is an ER-anchored enzyme, we sought to identify sites of interaction by immunofluorescence analysis of human liver tissue yet found no co-localization of BSEP and BACS (S10A Fig). Strikingly, we find that in differentiated HepaRG cells, a human hepatoma cell line with many hepatocyte-like characteristics missing in other liver cell lines [22], both BSEP and BACS are found in newly formed pseudo-canalicular structures (S10B and S10C Fig). This finding raises the possibility of a canalicular site of interaction between BSEP and one of the enzymes that catalyze the formation of its substrate—amino acid-conjugated bile salts.

### BSEP interacts with the membrane-cytoskeletal cross-linker radixin

Radixin (*RDX*) was identified as an interaction partner of BSEP by MYTH, co-IP / MS/MS and specific *in vitro* pulldown. This protein belongs to the ezrin/radixin/moesin (ERM) family and cross-links membrane proteins to the cytoskeleton [41]. The N-terminal FERM (4.1/ezrin/radixin/moesin) domain interacts with target membrane proteins, for example MRP2, and is only accessible if the protein is activated or the inhibiting C-terminus is removed [11,42]. The C-terminal ERM-associated domain (C-ERMAD) binds the actin cytoskeleton. Alternatively, ERM proteins can bind other scaffolding proteins, such as NHERF1/EBP50 [41]. In the MYTH assay (Fig 4A) a radixin fragment was produced that roughly corresponds to the N-terminal FERM domain. Looking at the co-IP results, radixin showed differential precipitation in the two detergents used. While there was no specific co-precipitation with Triton X-100, the complex of BSEP and radixin was stable in the presence of digitonin (Fig 3A). Immunoblot analysis of the co-IPs mirrored the results obtained from MS/MS analysis. With digitonin radixin appears in the presence of BSEP only, while with Triton X-100 it is also present in the control lanes, suggesting nonspecific binding in that detergent (Fig 3B). Pull-down analyses, as seen in Fig 4B, confirmed the interaction of BSEP with the N-terminal half of radixin (radixin_1-318_). Additionally, we could show that the C-terminal affinity tags used for purification of BSEP do not influence its interaction with radixin. After proteolytic removal of the BSEP affinity tags, pull-down assays show that radixin_1-318_ can bind BSEP, while full-length radixin does not display a signal as depicted in Fig 4C.

One likely protein interaction partner for BSEP could not be found in our approaches. The scaffolding protein NHERF1 is an interaction partner of several ABC-transporters in the liver, for example CFTR, MRP2 and MRP4 [13,43,44]. NHERF1, like radixin, is predominantly found at the apical membrane of hepatocytes [45] and was tested separately with MYTH, co-IP/ MS/MS and *in vitro* pull-down. We did not detect interaction of NHERF1 and BSEP in any of the three methods applied here.

## Discussion

Protein-protein interaction presents the basis of many cellular processes and also plays a pivotal role in the trafficking, localization and activity of membrane proteins. In the case of the bile salt export pump this has been shown for interactions that modulate its cycling at the canalicular membrane [8–10]. In the study presented here, we identified ten novel proteins that interact with BSEP using a three-sided approach.

**Fig 7:**
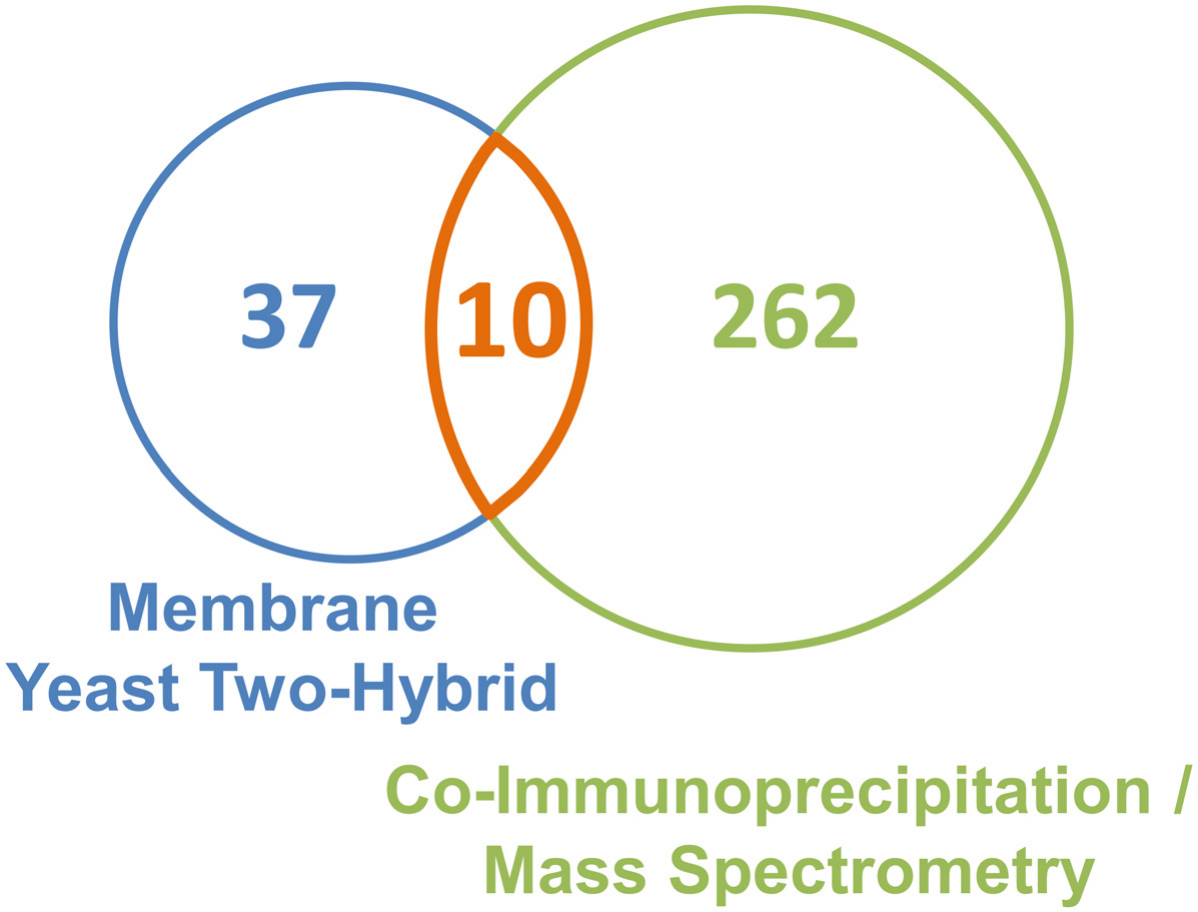
Venn diagram of potential BSEP interaction partners obtained with MYTH and co-IP / MS/MS. The numbers represent the potential BSEP interaction partners identified by MYTH and co-IP / MS/MS and the overlapping set of proteins found in both screens.

The first approach was used to screen for proteins that bind directly to BSEP. The MYTH assay probed BSEP interaction with soluble and membrane-associated liver proteins. Of the over 500 initial candidates 37 were verified as interaction partners after applying the appropriate controls (Fig 7). In contrast to currently available methods for human tissues, the MYTH assay has the advantage of detecting both stable and more transient interactions. The 27 additional BSEP interaction partners detected by MYTH, which were not found by co-IP / MS/MS, may represent these short-lived interactions (Table 1). On the other hand the host organism, the required fusion proteins, and the quality of the cDNA library limit the detection range.

Immunoprecipitation of BSEP from human liver and subsequent identification of interaction partners by MS/MS resulted in more than 200 BSEP-associated proteins (Fig 7). This approach allowed for identification of physiological interaction partners and proteins stably associated with the transporter via mediators. On the downside, the method is not suitable for reliable detection of transient interactions and the required use of detergent can disrupt physiologically relevant interactions. Consequently, we used two different, non-ionic detergents, digitonin and Triton X-100. By binding cholesterol [46] digitonin readily disrupts the cholesterol-rich canalicular membrane and was able to preserve the interaction of BSEP and radixin at the plasma membrane. A somewhat more stringent solubilization by Triton X-100, on the other hand, was required to access interactions in the secretory pathway.

The third method, *in vitro* pull-down, was used to confirm the interaction of BSEP with soluble proteins or soluble domains of identified interaction partners. This method requires soluble or solubilized proteins and detects stable interactions.

Applying the three presented approaches, which covered both *in vitro* methods as well as procedures for detecting physiological interactions in human liver, established the validity of the observed interactions. Proteins detected by an individual technique may still be relevant and require a different method for verification. Since methods for detection of PPI directly in human tissue are at present very limited, *in vitro* and yeast-based approaches help to cover the cases in which PPI cannot yet be screened and visualized *in vivo*.

The interaction partners identified in the early secretory pathway are likely involved in the topogenesis, sorting and quality control of BSEP. The TRAP complex, of which subunits alpha, gamma and delta have been found to associate with BSEP, assists in membrane protein topogenesis. Interaction of the nascent protein with the TRAP complex may stabilize certain orientations [26]. Influence on the sorting of secretory proteins has been shown for TRAM [27] and Bap31, for the latter in the sorting of CFTR [29]. Members of the REEP family influence the shape of ER membranes and processing of their target proteins [32] and may influence BSEP trafficking by locally altering ER membrane shape. RER1 is highly conserved across species and has been studied in more detail in yeast [47]. In combination with previous studies our findings indicate that Rer1 interacts with BSEP via its C-terminal transmembrane domain. Since several of the interacting proteins in the early secretory pathway are conserved among species, it is possible that yeast enabled an interaction in the MYTH assay with endogenous factors.

The enzymes that were found to interact with BSEP were the acyl-CoA synthetases BACS and VLACS. Their similarities, especially in the C-terminal 50 amino acids that were found to bind to BSEP, may point to a common binding site. BACS and VLACS are both catalyzing steps in the synthesis of BSEP substrates and may be involved in a regulatory feedback loop that modulates trafficking or modification of the bile salt transporter. In light of the results of protein-mediated taurocholate uptake in the ER [40], one might speculate that the proposed transport system is indeed BSEP, which would already in the ER catalyze the transport of bile acids. In contrast, the finding that BACS is localized at pseudocanalicular structures in differentiated HepaRG cells (S10C Fig) supports a canalicular site of interaction between BACS and BSEP. In contrast to the vast majority of existing canaliculi in adult human liver tissue, the pseudocanalicular structures in these cells are formed *de novo* during differentiation, and BACS may play some role in this process. We speculate that in the early stages of canalicular morphogenesis, a canalicular localization of BACS allows for the generation of conjugated bile salts in direct vicinity of their cognate transporter while later on this process is localized to the cytosolic side of the ER membrane.

Lastly, BSEP interacts with the FERM domain of the cross-linking protein radixin. Radixin has been shown to connect several membrane proteins to the cytoskeleton either directly or via adaptors. In the case of BSEP and MRP2 Wang *et al*. observed an influence of radixin expression on localization and transport efficiency in rat hepatocytes, where silencing of radixin led to retention of the transporters in subapical compartments [12].

In summary, with the complementary methods of membrane yeast two-hybrid, *in vitro* pull down assay and co-immunoprecipitation from human liver samples combined with MS/MS we screened for novel interaction partners of BSEP and identified ten proteins. These results define a network of interacting proteins involved in topogenesis, trafficking and functional regulation (Fig 5) and suggest that BSEP is tightly controlled within the cell with respect to localization and function. In addition to the factors in the early secretory pathway, radixin and the bile acyl-CoA synthetase have a possible role in the regulation of the transporter. How these interaction partners regulate BSEP in the physiological context remains to be investigated. Our work provides the foundation for further research on post-translational BSEP regulation by protein-protein interaction.

## Supporting Information

S1 Fig Uncropped immunoblot of Fig 3B anti BSEP. (PNG)

S2 Fig Uncropped immunoblot of Fig 3B anti Radixin. (PNG)

S3 Fig Uncropped immunoblot of Fig 3B anti Adaptin α. (PNG)

S4 Fig Uncropped immunoblot of Fig 3B anti BACS (*SLC27A5*). (PNG)

S5 Fig Uncropped immunoblot of Fig 4B anti BSEP. (JPG)

S6 Fig Uncropped immunoblot of Fig 4B anti Strep-tag. (JPG)

S7 Fig Uncropped immunoblot of Fig 4C anti BSEP. (TIF)

S8 Fig Uncropped immunoblot of Fig 4C anti Strep-tag. Immunoblot is flipped in the final figure.
(TIF)

S9 Fig MS data of interaction partners from all four co-IP experiments. (PNG)

S10 Fig Immunofluorescence staining of human liver cryosections and differentiated HepaRG cells for MRP2, BSEP, and BACS. (TIFF)

S1 Table Mass spectrometry data on identified BSEP interaction partners. Data from two independent measurements are shown. See inserted legend for further information.
(XLSX)
