# Supplementary figures and images for: Analysis of the Bile Salt Export Pump (ABCB11) Interactome Employing Complementary Approaches
Source: PLoS One. 2016 Jul 29;11(7):e0159778. doi: 10.1371/journal.pone.0159778 (PMC4966956; doi:10.1371/journal.pone.0159778)

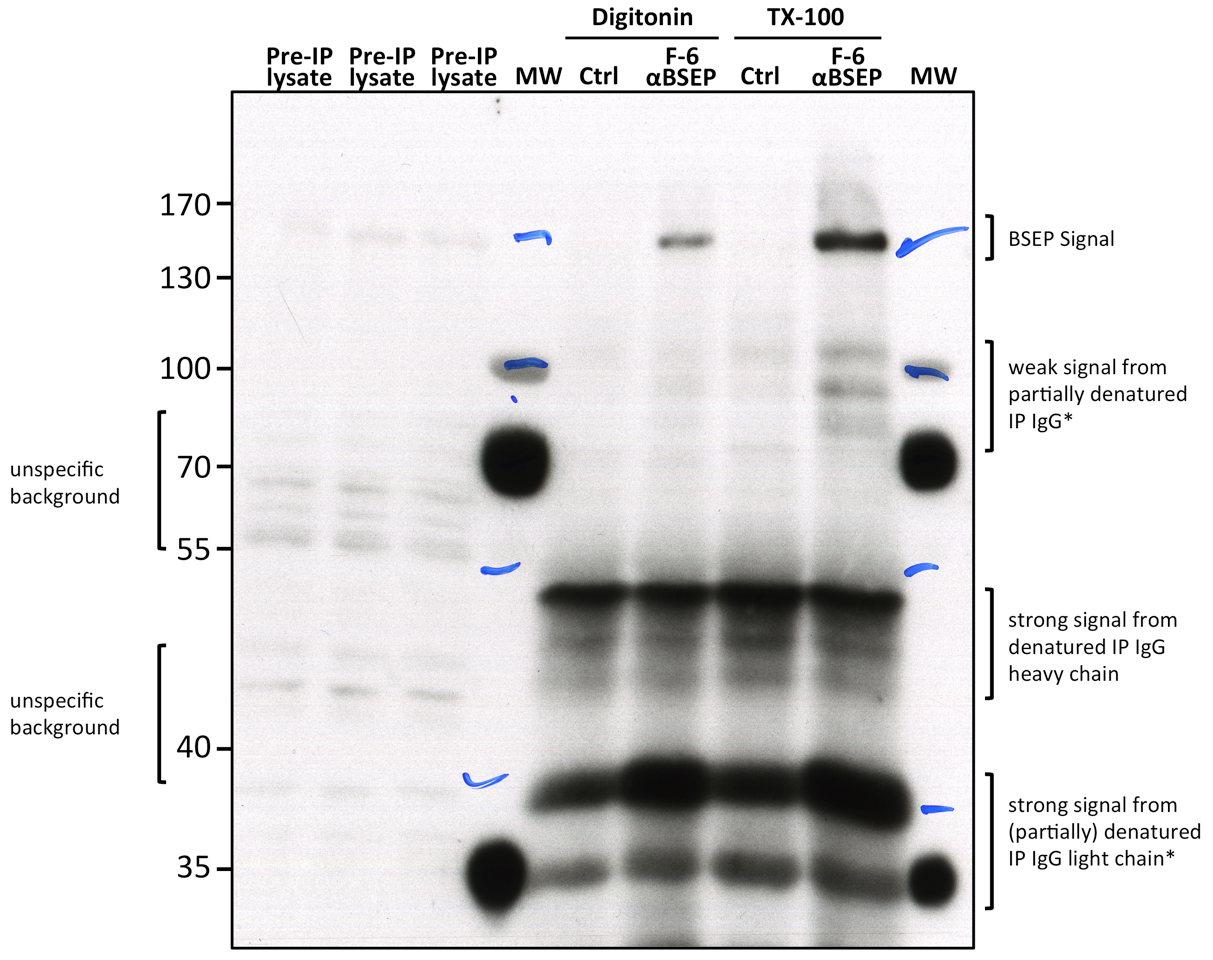

Supplement: S1 Fig — (PNG) [file pone.0159778.s001.png]

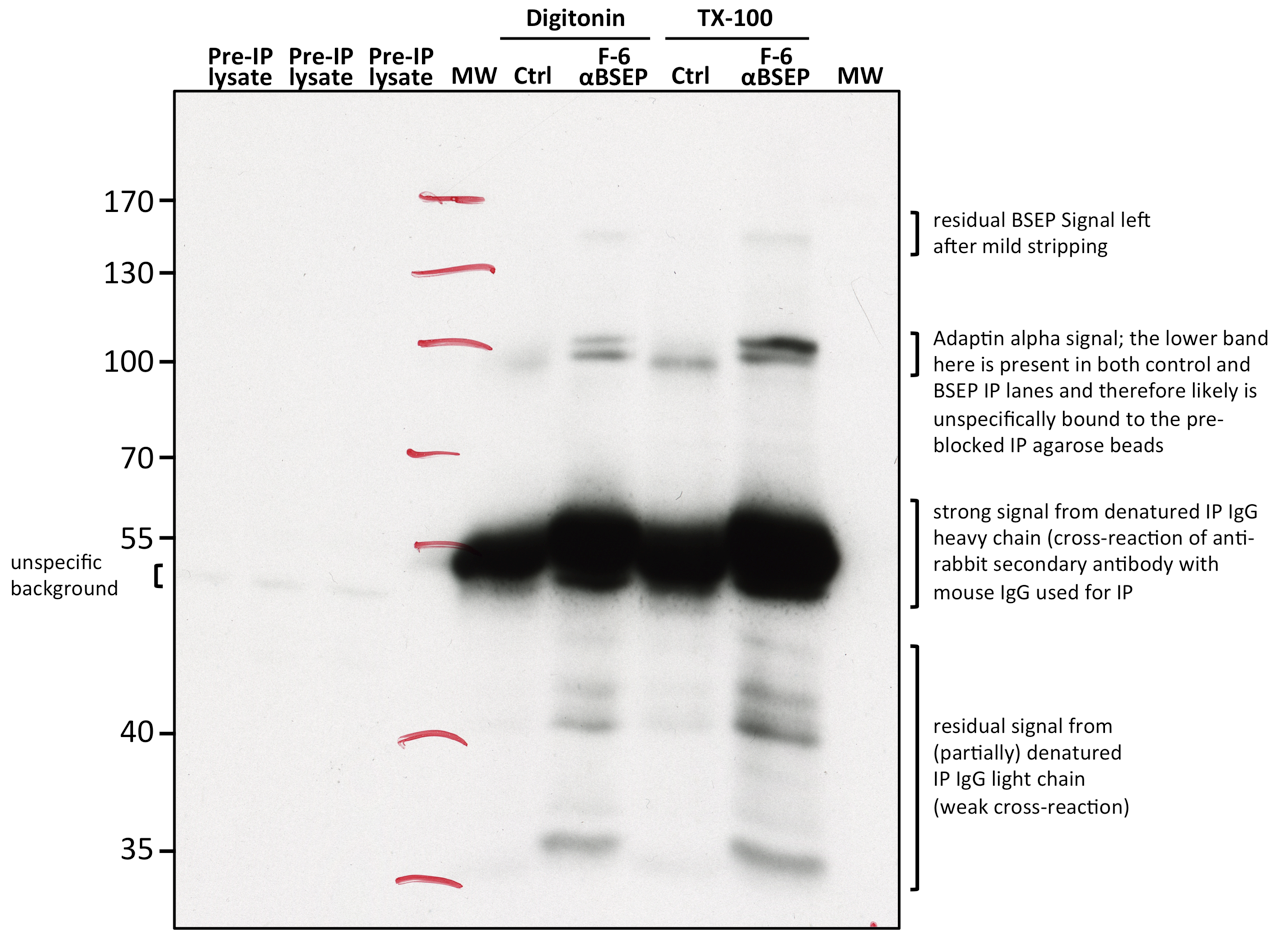

Supplement: S2 Fig — (PNG) [file pone.0159778.s002.png]

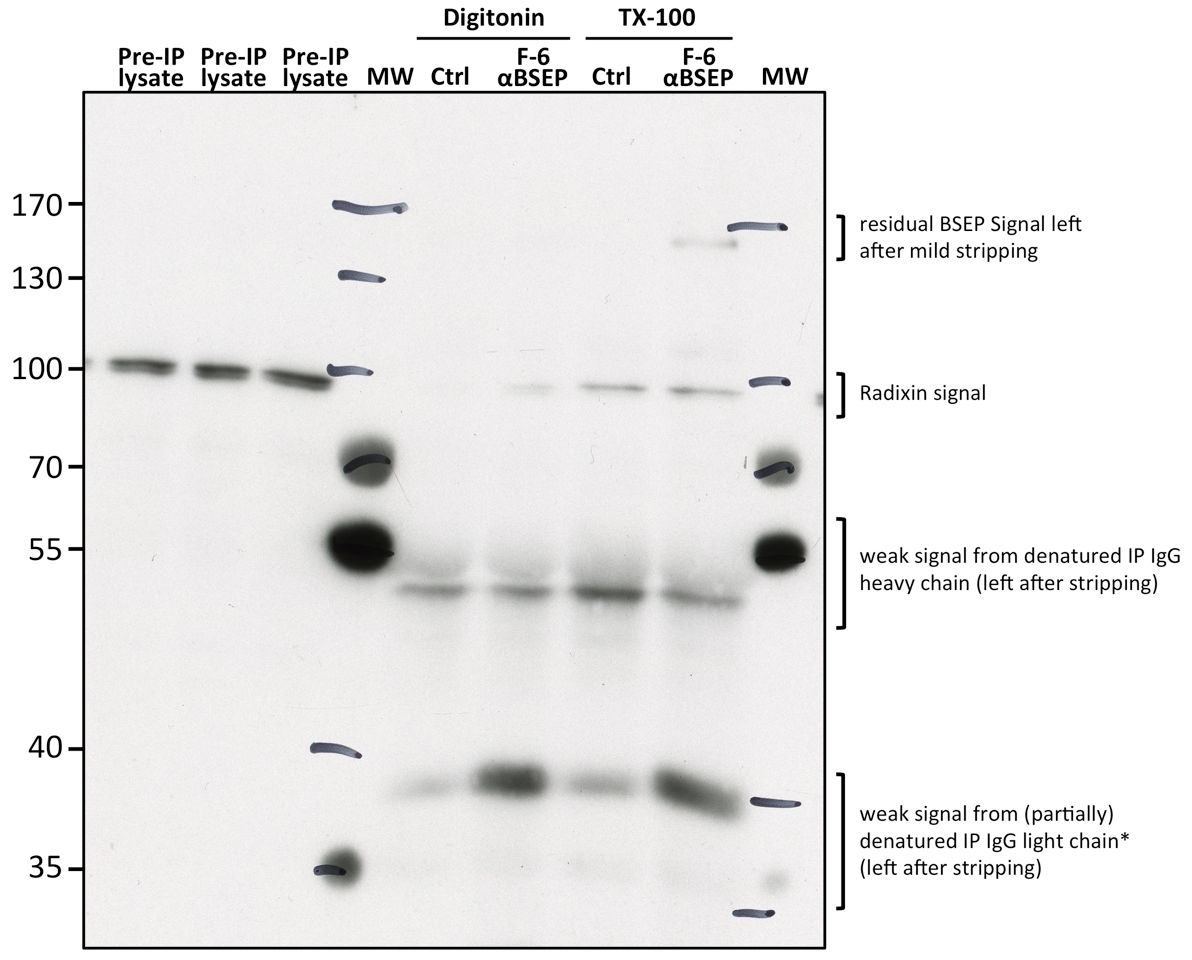

Supplement: S3 Fig — (PNG) [file pone.0159778.s003.png]

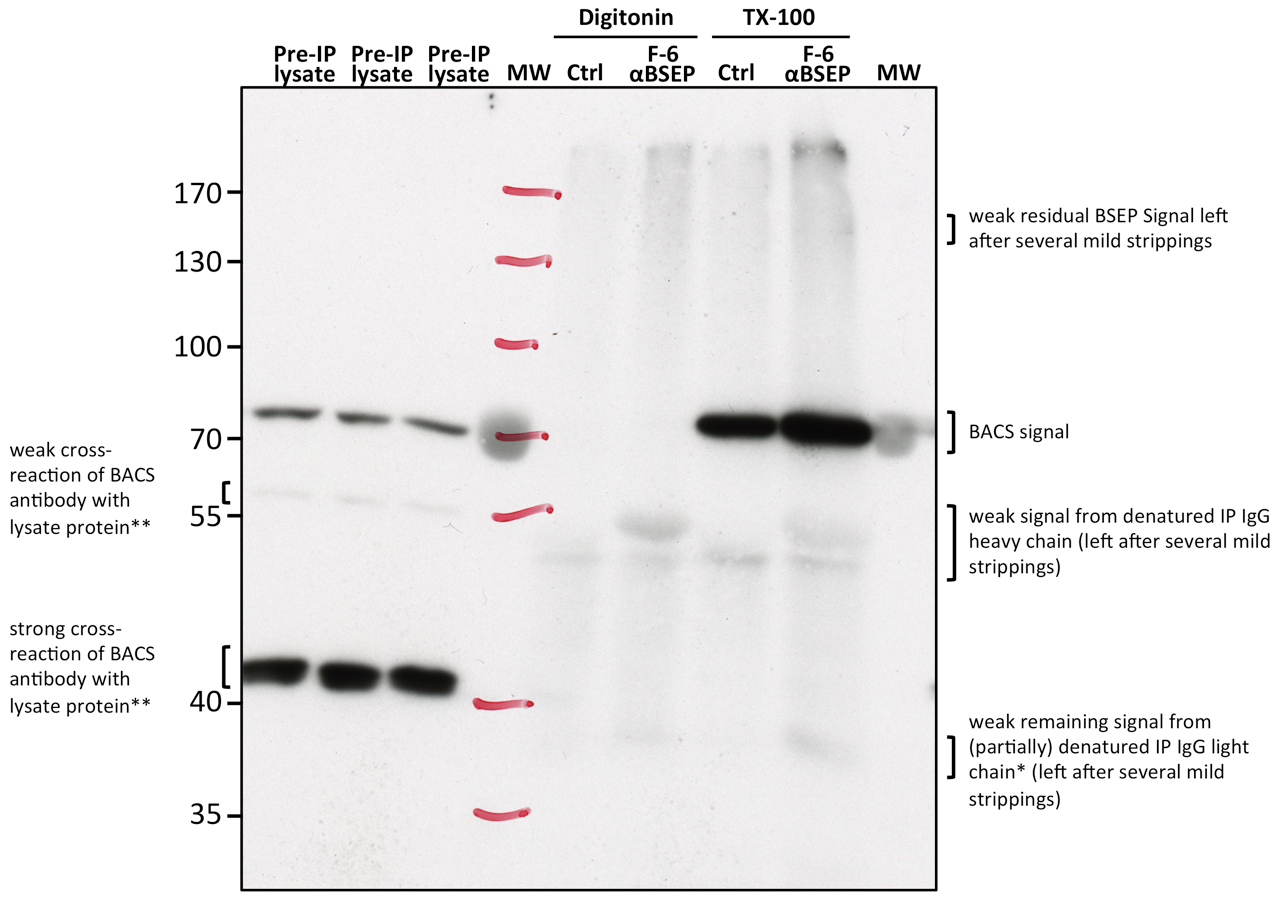

Supplement: S4 Fig — (PNG) [file pone.0159778.s004.png]

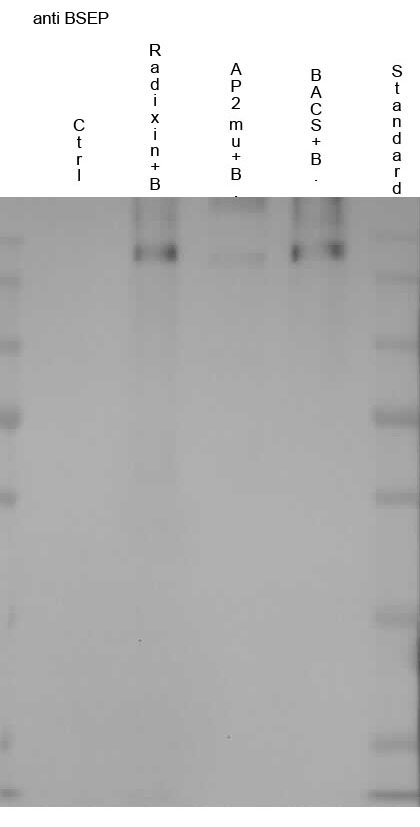

Supplement: S5 Fig — (JPG) [file pone.0159778.s005.jpg]

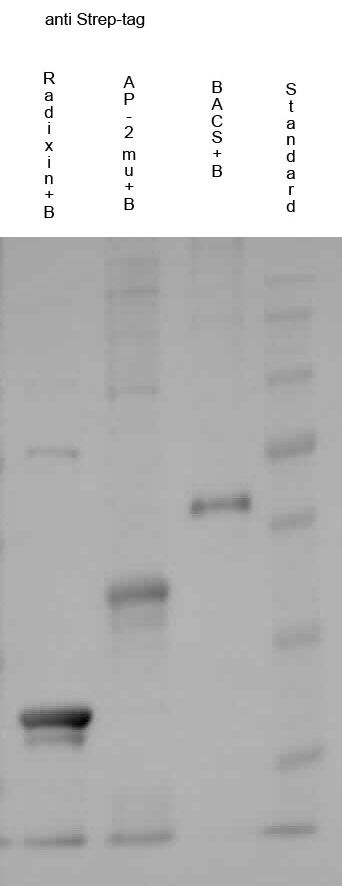

Supplement: S6 Fig — (JPG) [file pone.0159778.s006.jpg]

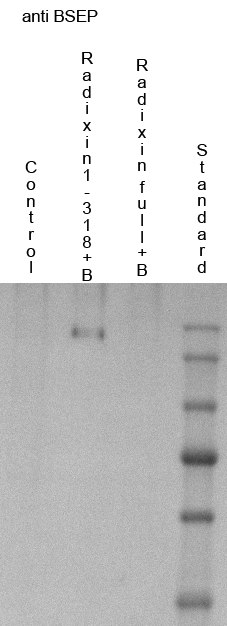

Supplement: S7 Fig — (TIF) [file pone.0159778.s007.tif]

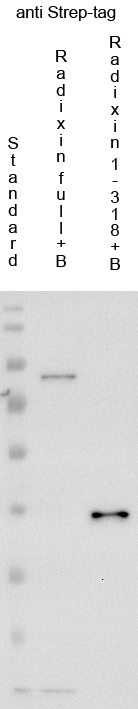

Supplement: S8 Fig — Immunoblot is flipped in the final figure. (TIF) [file pone.0159778.s008.tif]

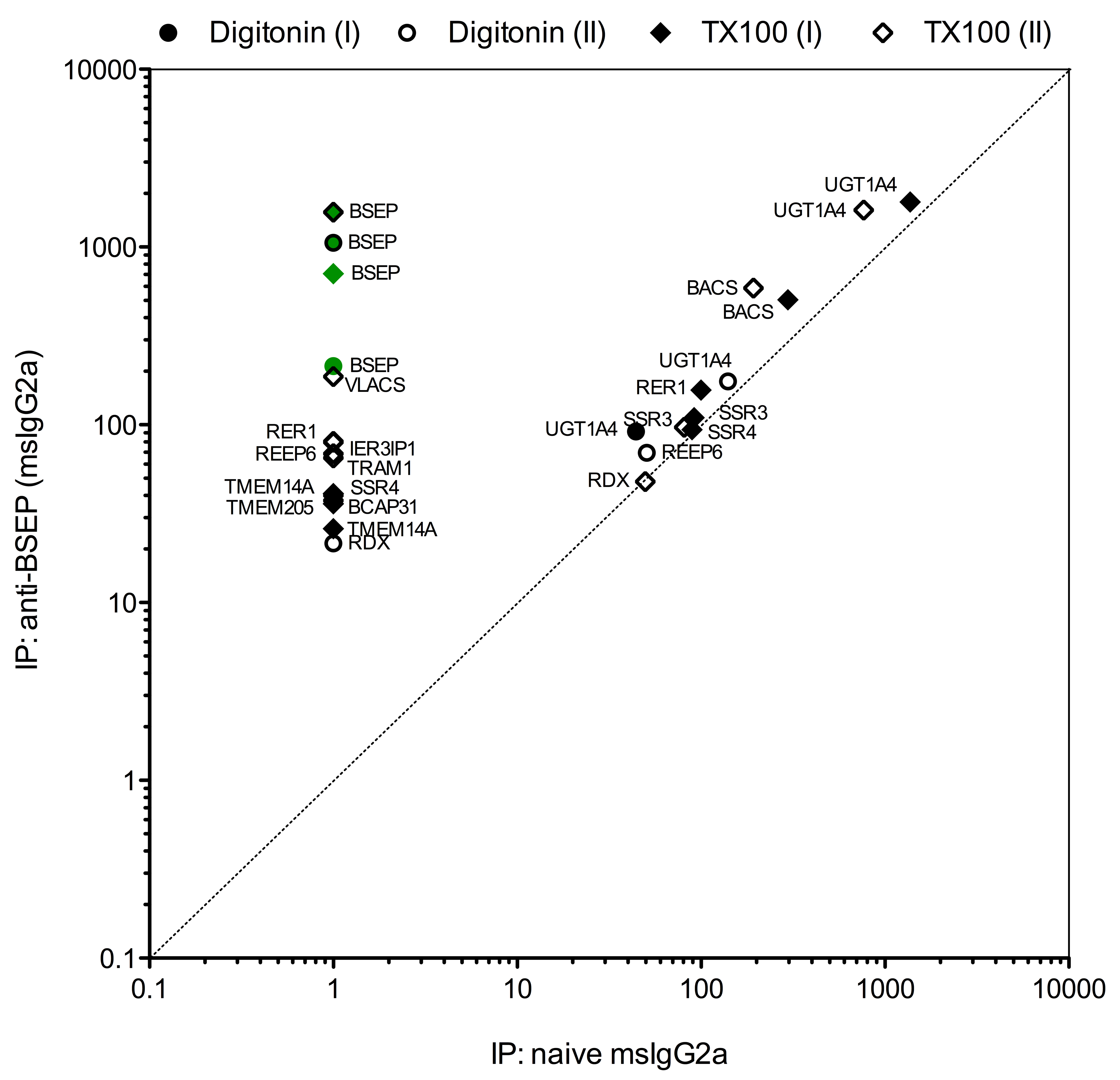

Supplement: S9 Fig — (PNG) [file pone.0159778.s009.png]

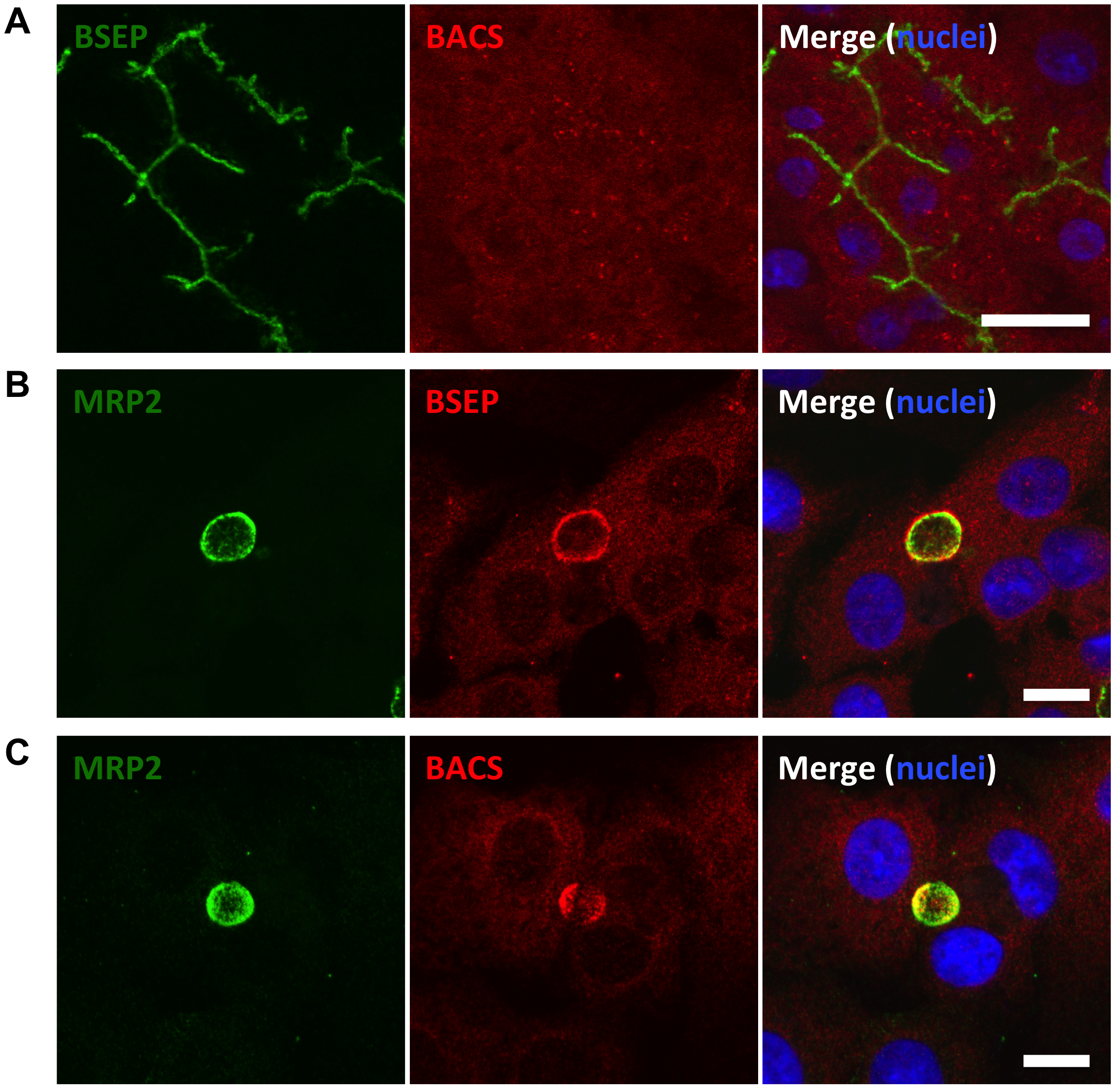

Supplement: S10 Fig — (TIFF) [file pone.0159778.s010.tiff]
